# Microarray analysis of MicroRNA expression in peripheral blood mononuclear cells of critically ill patients with influenza A (H1N1)

**DOI:** 10.1186/1471-2334-13-257

**Published:** 2013-06-03

**Authors:** Hao Song, Qi Wang, Yang Guo, Shunai Liu, Rui Song, Xuesong Gao, Li Dai, Baoshun Li, Deli Zhang, Jun Cheng

**Affiliations:** 1 MOA Key Laboratory of Animal Biotechnology of National Ministry of Agriculture, Institute of Veterinary Immunology, and Research Laboratory of Virology, Immunology & Bioinformatics, Division of Veterinary Microbiology & Virology, Department of Preventive Veterinary Medicine, College of Veterinary Medicine, Northwest A & F University, Yangling, Xi’an City, Shaanxi Province, 712100, China; 2 Institute of Infectious Diseases, Beijing Ditan Hospital, Capital Medical University, Beijing, 100015, China; 3 Beijing Key Laboratory of Emerging Infectious Diseases, Beijing, 100015, China; 4 Investigation Group of Molecular Virology, Immunology, Oncology & Systems Biology, Center for Bioinformatics, College of Life Sciences, Northwest A & F University, Yangling, Xi’an City, Shaanxi Province, 712100, China; 5 Department of Infectious Diseases, Beijing Ditan Hospital, Capital Medical University, Beijing, 100015, China

**Keywords:** Critically ill patient, 2009 H1N1 Influenza pandemic, miRNA, Host pathogen interaction, Systems biology

## Abstract

**Background:**

With concerns about the disastrous health and economic consequences caused by the influenza pandemic, comprehensively understanding the global host response to influenza virus infection is urgent. The role of microRNA (miRNA) has recently been highlighted in pathogen-host interactions. However, the precise role of miRNAs in the pathogenesis of influenza virus infection in humans, especially in critically ill patients is still unclear.

**Methods:**

We identified cellular miRNAs involved in the host response to influenza virus infection by performing comprehensive miRNA profiling in peripheral blood mononuclear cells (PBMCs) from critically ill patients with swine-origin influenza pandemic H1N1 (2009) virus infection via miRNA microarray and quantitative reverse-transcription polymerase chain reaction (qRT-PCR) assays. Receiver operator characteristic (ROC) curve analysis was conducted and area under the ROC curve (AUC) was calculated to evaluate the diagnostic accuracy of severe H1N1 influenza virus infection. Furthermore, an integrative network of miRNA-mediated host-influenza virus protein interactions was constructed by integrating the predicted and validated miRNA-gene interaction data with influenza virus and host-protein-protein interaction information using Cytoscape software. Moreover, several hub genes in the network were selected and validated by qRT-PCR.

**Results:**

Forty-one significantly differentially expressed miRNAs were found by miRNA microarray; nine were selected and validated by qRT-PCR. QRT-PCR assay and ROC curve analyses revealed that miR-31, miR-29a and miR-148a all had significant potential diagnostic value for critically ill patients infected with H1N1 influenza virus, which yielded AUC of 0.9510, 0.8951 and 0.8811, respectively. We subsequently constructed an integrative network of miRNA-mediated host-influenza virus protein interactions, wherein we found that miRNAs are involved in regulating important pathways, such as mitogen-activated protein kinase signaling pathway, epidermal growth factor receptor signaling pathway, and Toll-like receptor signaling pathway, during influenza virus infection. Some of differentially expressed miRNAs via in silico analysis targeted mRNAs of several key genes in these pathways. The mRNA expression level of tumor protein T53 and transforming growth factor beta receptor 1 were found significantly reduced in critically ill patients, whereas the expression of Janus kinase 2, caspase 3 apoptosis-related cysteine peptidase, interleukin 10, and myxovirus resistance 1 were extremely increased in critically ill patients.

**Conclusions:**

Our data suggest that the dysregulation of miRNAs in the PBMCs of H1N1 critically ill patients can regulate a number of key genes in the major signaling pathways associated with influenza virus infection. These differentially expressed miRNAs could be potential therapeutic targets or biomarkers for severe influenza virus infection.

## Background

In 2009, human infection with novel swine-origin influenza A (H1N1) virus became a health burden throughout the world. The H1N1 virus spread rapidly to countries worldwide, leading the World Health Organization (WHO) to declare on 11 June 2009 the first influenza pandemic in more than 40 years [1].

Like other viruses, influenza virus relies on host cellular processes throughout its replication cycle. Various strategies have been used to characterize host factors involved in influenza virus infection to better understand the molecular mechanisms of viral pathogenesis. These strategies include yeast two-hybrid analysis [2], genome-wide RNA interference (RNAi) screen [3-5], and integrative analysis combining several different approaches [6,7]. Hundreds of host proteins have been identified and a physical, regulatory, and functional map of host-influenza interactions has been drawn, which shows the global perspective of virus infection and uncovers the complex host-pathogen relationships. However, the concrete mechanism is still unclear; more studies relevant to influenza virus are still needed.

MicroRNAs (miRNAs) are small (~22 nucleotides long), single-stranded non-coding RNAs that mediate posttranscriptional silencing of target genes. In animals, miRNAs usually bind to complementary sites in the 3′ untranslated region (UTR) of specific target genes, resulting in inhibited protein expression and induced target mRNA degradation [8]. MiRNAs have emerged as key regulators of diverse biological processes, including development [9], cancer [10], immune response [8,11] and so on. Special miRNAs have been reported to participate in regulating cross-talk between the host and the pathogen in viral infections and have a major function in viral pathogenesis [12-14]. For influenza virus, differential expression of cellular miRNAs have been found both in avian influenza virus-infected chickens [15,16] and reconstructed 1918 influenza virus [17] or the highly pathogenic avian influenza H5N1 virus [18] infected mice. Several cellular miRNAs, such as miR-323, miR-491, miR-654, and Let-7c have recently been reported to inhibit H1N1 influenza A virus replication by downregulating the viral gene expression in infected MDCK or A549 cells [19,20]. In addition, temporal- and strain-specific host miRNA molecular signatures have been demonstrated in human A549 cells infected with swine-origin influenza pandemic H1N1 (2009) and highly pathogenic avian-origin influenza H7N7 (2003) [21]. However, it is still unclear whether miRNAs also play an important role in human being infected with influenza virus, especially critically ill patients caused by influenza virus infection.

Human peripheral blood mononuclear cells (PBMCs) provide an important source for clinical diagnosis and pathogenesis discovery. In contrast to target tissue biopsy, blood is not limited by restricted access to target tissues. Blood is a highly dynamic environment, which is another advantage. Blood has been proposed as a ‘sentinel tissue’ that reflects disease progression in the body [22]. The leukocytes can interact and communicate with practically every tissue so that these cells have rich information regarding inflammation and immune responses. Gene expression profiling in peripheral blood has been used to describe the pathogenesis of infectious diseases, including influenza, and to discover unique signatures of disease or to identify novel drug targets for treatment [22-33]. Influenza A virus can infect and replicate in human primary dendritic cell (DC), macrophages, and natural killer cells [34-36]. Therefore, it is appropriate to use PBMC for gene expression profiling, and it holds great promise for clinical diagnosis and research [22].

Although multiple signaling pathways and various cellular factors have been associated with influenza virus infection, the function of the miRNAs of PBMCs is still poorly understood. In the current study, we used both miRNA microarray and quantitative reverse-transcription polymerase chain reactions (qRT-PCR)-based approaches to assess miRNA expression in PBMCs from the critically ill patients with H1N1 infection, and found some differentially expressed miRNAs that can be highly related to influenza virus infection. We subsequently constructed a direct gene interaction network to illustrate the interaction mechanism of these miRNA targets with each other via protein-protein interaction during influenza virus infection. This network revealed potential important functions that miRNAs have in host and pathogen interactions, and provided several directions for further study. We then validated several hub genes in the network using the qRT-PCR method and demonstrated that the hub genes, which are highly important during influenza virus infection, can be modulated by multiple miRNAs.

## Methods

### Ethics statement

This study was approved by the Beijing Ditan Hospital Ethics Committees, and informed consent was obtained from subjects involved at the time of sample collection. All volunteers provided written informed consent for sample collection and subsequent analysis.

### Patients and control individuals

From September 2009 to November 2009, a total of 299 confirmed cases of human infection with the novel strain H1N1 were admitted to the intensive care unit (ICU) of Beijing Ditan Hospital in China. We classified the patients according to the case definition developed by the Ministry of Health of China. The symptoms in severely ill patients included: (1) sustained high fever over 3 d; (2) violent cough with purulent sputum or blood in sputum and chest pain; (3) increased respiratory frequency, dyspnea, and cyanosis; (4) altered mental status, such as unresponsiveness, lethargy, restlessness, or seizures; (5) severe vomiting or diarrhea with dehydration; (6) signs of pneumonia in chest X-ray or computerized tomography scan; (7) rapid increase in cardiac enzymes including creatine kinase (CK) or creatine kinase isoenzyme (CK-MB), and (8) aggravation of basic illness.

Critical cases were defined when one of the following conditions occurred: (1) respiratory failure; (2) septic shock caused by severe infection; (3) multiple organ dysfunction syndrome, or (4) requirement of intensive care [37]. The diagnoses were confirmed using the specific RT-PCR protocol developed by the Center for Prevention and Disease Control in Atlanta, Georgia, USA, and recommended by WHO for Human Influenza A/H1N1/2009 [38]. Thirteen healthy donors with no recent illness or treatment for a chronic medical condition and diagnosed as negative to influenza A/H1N1 using the specific RT-PCR protocol were included as control group.

### RNA isolation and quality control

Blood samples were collected in EDTA-treated tubes as soon as the patients were admitted to the ICU. PBMCs were isolated by standard Ficoll density gradient centrifugation and stored in RNAlater (Ambion) at −80°C before RNA isolation.

Total RNA was isolated using the mirVana miRNA PARIS kit (Ambion), according to the protocol of the manufacturer. RNA concentration and RNA integrity were determined by capillary electrophoresis on an Agilent 2100 Bioanalyzer (Agilent Technologies); only the samples with RNA integrity number > 7 were used. RNA samples were stored at −80°C until further processing.

### MiRNA expression profiling

The Agilent human miRNA microarrays (version 3.0, based on Sanger miRBase version 13.0) [39] were used to compare the expression profiles of critically ill patients (n = 5) and healthy controls (n = 3). The samples used for miRNA expression profiling were randomly selected from the two groups. Total RNA (100 ng) from each sample was used as inputs for labeling via Cy3 incorporation. After hybridization and washing, microarray slides were scanned with Aligent Microarray Scanner (Agilent, Santa Clara, CA, USA). Scans were performed at 5 μm resolution and dye channel was set to green (PMT100, PMT5). Labeling and hybridization were performed at the Shanghai Biochip Company, according to the protocols in the Agilent miRNA microarray system. Microarray images were analyzed with Feature Extraction Software (Agilent, Santa Clara, CA, USA). The signal after background subtraction was exported directly into the GeneSpring GX10 software (Agilent Technologies, Santa Clara, CA) for quantile normalization. The mean normalized signal from biological replicates was used for comparative expression analysis. For the filtering step, the features (miRNA) whose percentage of detection is 100%, under at least one experimental condition, are retained for further analysis. Significance analysis of Microarrays [40] software was used to determine differentially expressed miRNAs between patient and control groups. Gene Cluster 3.0 [41] and Java TreeView software [42] were used to perform differentially expressd miRNA hierarchical cluster analysis and visualization.

### Microarray data submission

The microarray data submission for human arrays is MIAME compliant. The raw and normalized microRNA data have been deposited in NCBI’s Gene Expression Omnibus (GEO) database [43] and are accessible through GEO Series accession number GSE24956.

### QRT-PCR

QRT-PCR of microRNAs was performed using Taqman miRNA assays (Applied Biosystems, Foster City, California), according to the instructions of the manufacturer, with the 7500 real-time PCR system (Applied Biosystems, Foster City). The assays were performed for nine miRNAs (has-miR-146b-5p, has-miR-148a, has-miR-150, has-miR-31, has-miR-155, has-miR-29a, has-miR-29b, has-miR-342-5p, and has-miR-886-3p) in larger sample sets obtained from PBMCs of eleven critically ill patients with H1N1 infection and thirteen healthy controls. The expression level of the small nuclear RNU44 (RNA, U44 small nuclear) was used as the normalization control. All assays were performed in quadruplicate. Relative expression levels were calculated using the 2^-ΔΔCt^ method. Data quantification was calculated via *t*-test between the patient and control groups using the RealTime StatMiner® Software (Integromics, Spain). Two-tailed P values ≤ 0.05 were considered statistically significant for differences.

QRT-PCR of mRNAs was measured using an ABI Prism 7500 (Applied Biosystems, USA) and SYBR® Premix Ex Taq™ II (Takara, Japan) according to the instructions of the manufacturer. A total of 0.5 μg of RNA from each sample was used to generate cDNA as templates by RT with the PrimeScript RT reagent kit (Takara, Japan). Primer pairs used for real-time PCR were shown in Table 1. The results of the qRT-PCR were normalized to β-actin expression. All assays were performed in triplicate. Relative expression levels were calculated using the 2^-ΔΔCt^ method. Data quantification was calculated via *t*-test between the patient and control groups using the RealTime StatMiner® Software. Two-tailed P values ≤ 0.05 were considered statistically significant.

**Table 1:** qRT-PCR primers used in the study

**Gene**	**Sequence**
**TP53**	5′-CCTCCTCAGCATCTTATCCGAGTG-3′(forward)
	5′-CCAACCTCAGGCGGCTCATAG-3′(reverse)
**CASP3**	5′-AGAACTGGACTGTGGCATTGAG-3′ (forward)
	5′-GCTTGTCGGCATACTGTTTCAG-3′(reverse)
**JAK2**	5′-CGGTATGACCCTCTACAGGACAAC-3′(forward)
	5′-AGATTACGCCGACCAGCACTG-3′(reverse)
**IL-10**	5′-TGCTGGAGGACTTTAAGGGTTACC-3′ (forward)
	5′-TGATGTCTGGGTCTTGGTTCTCAG-3′(reverse)
**MX1**	5′-ACAATCAGCCTGGTGGTGGTC-3′ (forward)
	5′-TCAAGATTCCGATGGTCCTGTCTC-3′(reverse)
**TGFBR1**	5′-GCTGTGAAGCCTTGAGAGTAATGG-3′ (forward)
	5′-GATGCCTTCCTGTTGACTGAGTTG-3′(reverse)
**MAPK14**	5′-GGAGGTGCCCGAGCGTTAC-3′ (forward)
	5′-TGGACTGAAATGGTCTGGAGAGC-3′(reverse)
**β-actin**	5′-TGGCACCCAGCACAATGAA-3′ (forward)
	5′-CTAAGTCATAGTCCGCCTAGAAGCA-3′(reverse)

### Receiver operating characteristic (ROC) analysis

ROC curves were established to evaluate the diagnostic value of differentially expressed miRNAs for differentiating between critically ill patients and controls using Graphpad Prism software (California, USA). QRT-PCR data of the nine differentially expressed microRNAs were used for analysis. A P value of less than 0.05 was considered statistically significant. The ROC analysis tool was used to determine the sensitivity and specificity of each possible cut-off score. The cut-off score yielding the highest sum of specificity and sensitivity was used as optimal cut-off score.

### MiRNA target prediction

Different algorithms were used for miRNA target prediction, including miRanda [44], TargetScan 5.1 [45], miRDB [46], RNA22 [47], PICTAR5 [48] and miRwalk [49]. Only miRNA target genes identified by at least three of these algorithms were considered.

Thus far, a few parts of important miRNA target genes were validated in numerous studies. However, most miRNA target genes were still not validated by experiments. We obtained the validated target gene set of these differentially expressed miRNAs from miRwalk database.

### Protein-protein interaction

In our study, we used the protein-protein interactions from the STRING database [50], which integrates and weighs information from numerous sources, including conserved neighborhood, gene fusions, phylogenetic co-occurrence, co-expression, database imports, large-scale experiments, and literature co-occurrence [51]. The scores higher than 0.7 will be considered as high confidence [51], thus, we used the interactions with combined scores higher than 0.7 for further analysis.

### Enrichment analysis and network construction

DAVID [52], a functional annotation tool, was used to analyze the enriched KEGG [53] and REACTOME [54] pathways with default settings. The integrative network of miRNA-mediated host-influenza virus protein interactions was drawn using Cytoscape [55].

## Results

### Demographic and laboratory findings of the patients

Eleven critically ill patients with no underlying diseases were included in the study. All patients were presented with influenza-like syndrome and met the diagnostic criteria of critical case. Their mean ± SD age was 30.91 ± 8.1 years; eight patients were male and three were female. The levels of body mass index (BMI) were all greater than 25 kg/m^2^. Four of the patients were cured with noninvasive ventilation, and tracheal intubation was performed in the other seven patients. The CT scan showed that the pulmonary lesions of all patients rapidly progressed. The Mean ± SD white blood cells were 6.31 ± 3.66 mm^3^. The laboratory findings of the patients at the time of sample collection are summarized in detail in Table 2.

**Table 2:** Laboratory findings on collection day

**Variable**	**Value**
**Days from in accident to in hospital**	
**Mean±SD—d**	5.19 ± 1.94
**Leukocyte count**	
**Mean count—per mm**^**3**^	6.31 ± 3.66
**Lymphocyte count**	
**Mean count—per mm**^**3**^	1.57 ± 3.10
**Creatine kinase**	
**Mean±SD—U/liter**	871.3 ± 1254.02
**>200 U/liter — no./total no. (%)**	6/11(54.5)
**Creatine kinase MB fraction**	
**Mean±SD—U/liter [total no.]**	35.4 ± 18.16 [10]
**>25 U/liter—no./total no. (%)**	8/10 (80.0)
**Lactate dehydrogenase—U/liter**	
**Mean±SD—U/liter**	611.12 ± 368.81
**C-reactive protein >10 mg/liter — no./total no. (%)**	
**Mean±SD—U/liter**	133.07 ± 56.47
**Hydroxybutyrate dehydrogenase**	
**Mean±SD—U/liter**	607.91 ± 331.77

### Significantly differentially expressed miRNAs differentiate H1N1 critically ill patients from healthy control samples

**Figure 1:**
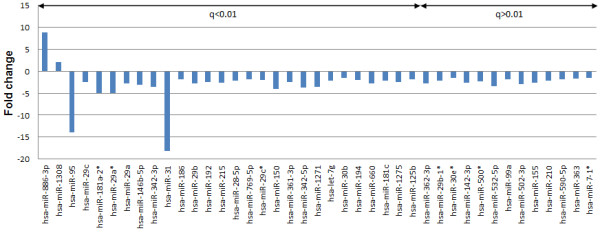
**Differentially expressed microRNAs between patients and control groups.** The diagram shows the fold changes of miRNAs that passed the filtering criteria using SAM software between the two groups in the analysis. FDR lower than 0.05 and fold change higher than 1.5 was set as standard. Up-regulated and down-regulated miRNAs are indicated by bars above and below the horizontal axis respectively. The q-values are indicated above the bars.

**Figure 2:**
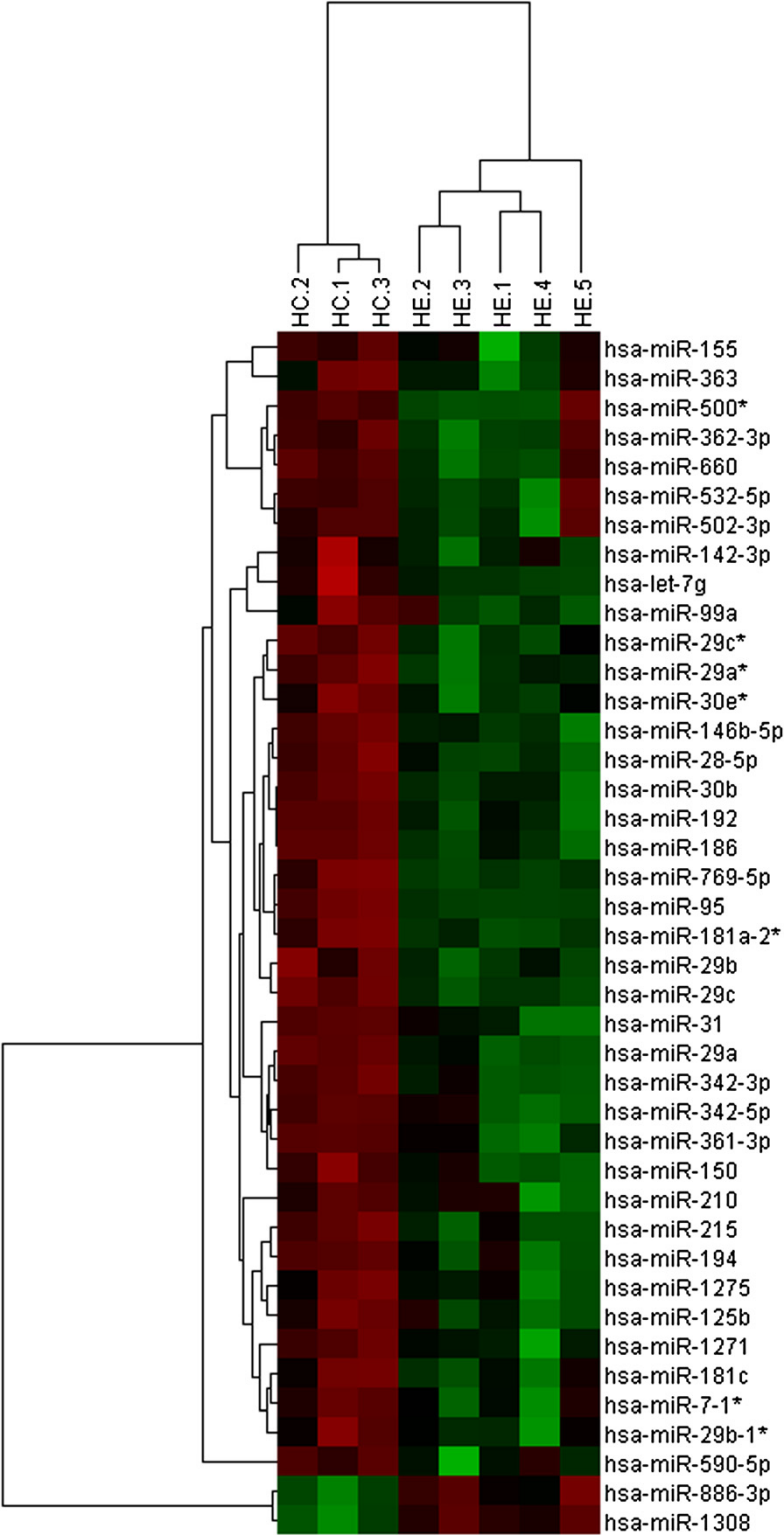
**A hierarchical clustering tree map comparing expression of miRNAs between patient (HE) and healthy control (HC) samples.** The clustering was performed on standardized log2 signals for the differentially expressed miRNAs, using Pearson correlation and average linkage clustering method. Green or red bars denote that the level of specific microRNA was decreased or increased in samples.

We performed a series of microarray profiling of cellular miRNAs in PBMCs from critically ill patients infected with H1N1 influenza virus and healthy controls to identify systematic differences in miRNA expression patterns during influenza virus infection. Forty-one human miRNAs were significantly differentially expressed between H1N1 critically ill patients and healthy controls, with false discovery rate lower than 0.05 and fold change higher than 1.5 (Figure 1). The cluster analyses revealed complete separation of the patient and control groups based on the expression profiles of the differentially expressed miRNAs (Figure 2).

### QRT-PCR validation of differentially expressed miRNAs and ROC analysis

**Figure 3:**
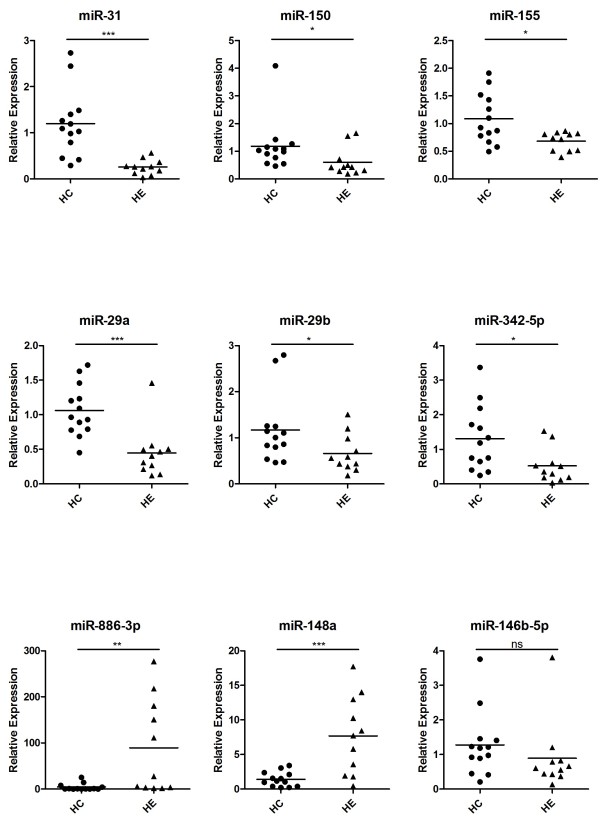
**qRT- PCR analysis of 9 miRNAs expression in the PBMCs from patients with H1N1 (n = 11) and normal controls (n = 13).** qRT-PCR analysis of 9 miRNAs expression in the PBMCs from H1N1 critically ill patients (n = 11;8 male 3 female; age 31 ± 8 y [mean±SD]) and healthy controls (n = 13; 9 male 4 female; age 29 ± 5 y [mean±SD]). The relative expression levels were normalized to the expression of RNU44. HE: H1N1 critically ill patients HC: healthy controls. * P < 0.05; **P < 0.01; ***P < 0.005.

**Figure 4:**
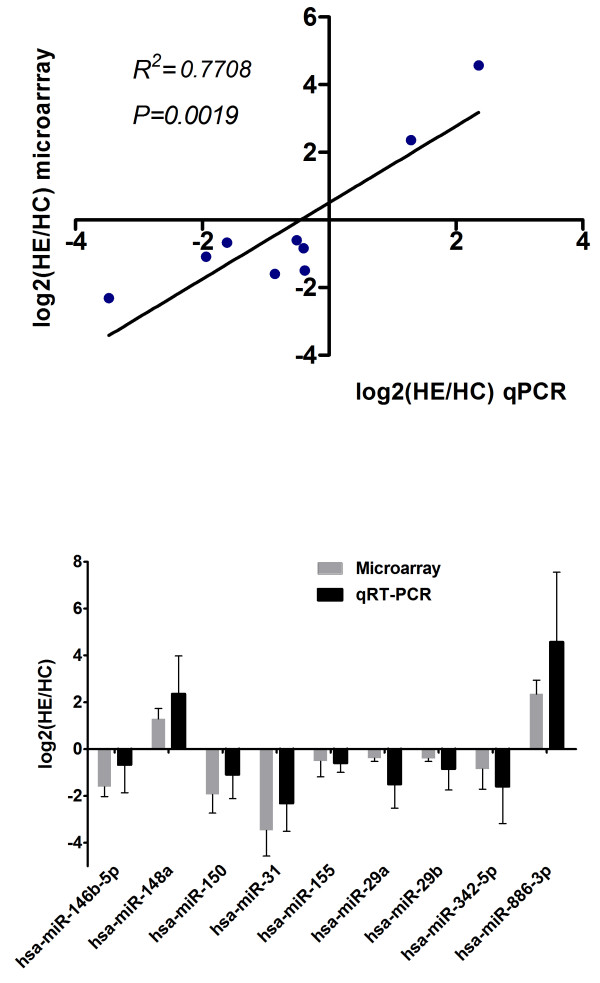
Comparison of miRNA expression levels obtained by miRNA microarray and Taqman qRT-PCR analysis.

The microarray data were validated by performing, qRT-PCR for nine miRNAs, including hsa-miR-146b-5p, hsa-miR-148a, hsa-miR-150, hsa-miR-31, hsa-miR-155, hsa-miR-29a, hsa-miR-29b, hsa-miR-342-5p, and hsa-miR-886-3p. We also considered hsa-miR-148a, which has an obvious fold change, but filtered by statistics test, and was proven highly important in previous studies. Subsequently, we used scatter plot to represent the relative expression levels of these nine miRNAs (Figure 3). The qRT-PCR results were in accordance with the miRNA microarray results. The expression of hsa-miR-150, hsa-miR-31, hsa-miR-155, hsa-miR-29a, hsa-miR-29b, hsa-miR-342-5p, and hsa-miR-146b-5p were present in lower abundance, whereas hsa-miR-148a and hsa-miR-886-3p were present in higher abundance in PBMCs from critically ill patients infected with H1N1 influenza virus than that from healthy controls. This result indicates a positive correlation between the quantities of transcripts measured by both microarray and qRT-PCR assay (Figure 4).

**Figure 5:**
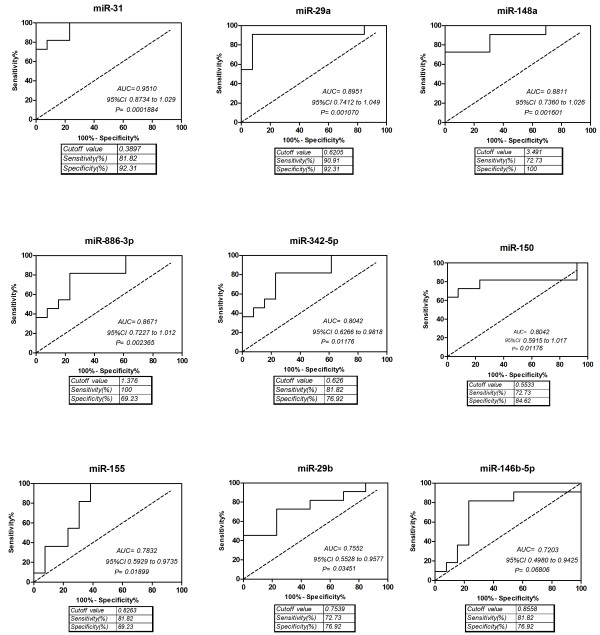
ROC curve analysis using differentially expressed miRNAs for discriminating critically ill patients.

ROC curve analyses revealed that miR-31, miR-29a and miR-148a were valuable biomarkers for differentiating critically ill patients from controls: miR-31 yielded an AUC (the areas under the ROC curve) of 0.9510 (95% CI: 0.8734–1.029; P = 0.0001884) with 81.82% sensitivity and 92.31% specificity in discriminating critically ill patients; miR-29a yielded AUC of 0.8951 (95% CI: 0.7412–1.049 P = 0.0001070) with 90.91% sensitivity and 92.31% specificity in discriminating critically ill patients, and miR-148a yielded AUC of 0.8811 (95% CI: 0.7360–1.026 P = 0.001601) with 72.73% sensitivity and 100% specificity in discriminating critically ill patients(Figure 5). However, miR-146b-5p could not discrimiate critically ill patients effectively due to the P value of ROC analysis was higher than 0.5(Figure 5). The result was consistent with the qRT-PCR result (Figure 3). The expression level of miR-146b-5p was only slightly decreased in critically ill patients compared to controls with no significant difference (P > 0.05).

### MiRNA target prediction and qRT-PCR validation

Numerous studies showed that miRNAs can influence gene expression by causing translational repression or mRNA degradation. This dysregulation can alter several downstream pathways and manifest effects. Therefore, miRNA gene target predictions from miRanda, Targetscan, miRDB, RNA22, PICTAR5, and miRwalk [44,45,47-49,56] were performed in our study. A total of 12,117 targets with 55,838 interactions were predicted.

Interactions between proteins provide a basis for most biological processes in an organism. The topological analysis can help obtain important information in the network formed by interacting proteins. Thus, in this study, we used the protein-protein interaction data from the STRING database [50] to construct the network of the target genes of the differentially expressed miRNAs to identify several ‘hub’ nodes (the high connected proteins), which have an important function in influenza virus infection. This study will help in the understanding of the potential functions of the differentially expressed miRNAs.

**Figure 6:**
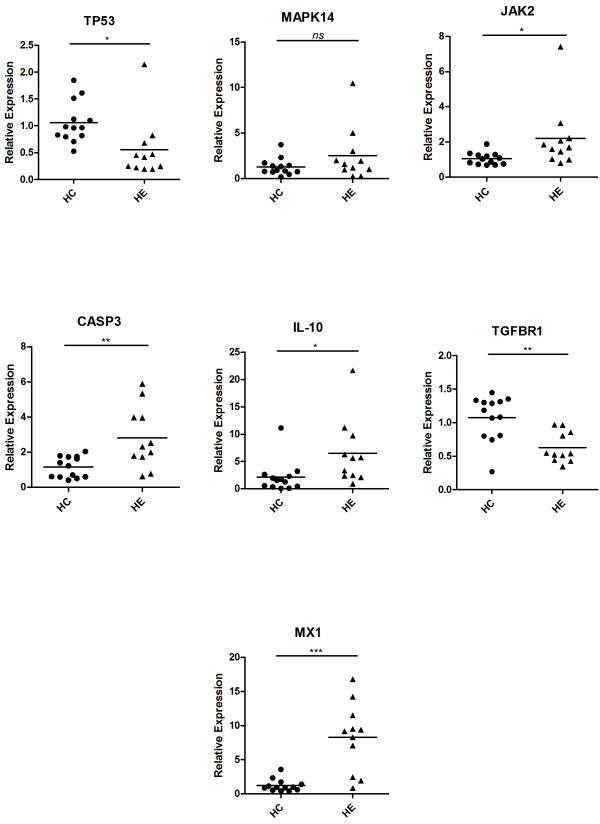
**qRT- PCR analysis of 6 mRNAs expressed in the PBMCs from patients with H1N1 (n = 11) and normal controls (n = 13).** The relative expression levels were normalized to the expression of β-actin. HE: H1N1 critically ill patients HC: healthy controls. * P < 0.05; **P < 0.01; ***P < 0.005.

QRT-PCR was performed for these ‘hub’ nodes expressed in the PBMCs from H1N1 patients (n = 11) and normal controls (n = 13), including tumor protein p53 (TP53, degree: 553), mitogen-activated protein kinase 14 (MAPK14, degree: 201), Janus kinase 2 (JAK2, degree: 197), caspase 3 apoptosis-related cysteine peptidase (CASP3, degree: 158), interleukin 10 (IL-10, degree: 112), transforming growth factor beta receptor 1 (TGFBR1, degree: 67), and myxovirus resistance 1 (MX1, degree: 32). We also used scatter plot to represent the relative expression levels of these seven mRNAs (Figure 6). The expression levels of JAK2, CASP3, IL-10, and MX1 significantly increased, whereas TP53 and TGFBR1 significantly decreased in PBMCs from critically ill patients infected with H1N1 influenza virus than that from healthy controls. Only a slight increase in the MAPK14 expression level was observed in PBMCs from critically ill patients with no significant difference.

### Integrative analysis of influenza virus-related miRNA-mRNA regulatory network

Like all viruses, influenza virus relies on the cellular machinery of the host to support their life cycle. Tokiko Watanabe et al. [57] summarized 1,449 cellular genes identified to date as important for influenza virus replication from several RNAi-based genome-wide screening experiments. Identifying the host functions co-opted for viral replication is of interest for the understanding of the mechanisms of the virus life cycle and to find valuable targets of differentially expressed miRNAs in our study. We obtained the data of virus-host interactions from previous studies [6,7], which can provide more insights into the molecular mechanism of diseases at systematic level.

Functional enrichment analysis (P < 0.05) performed to these cellular genes revealed various over-represented pathways, including the MAPK signaling pathway, Toll-like receptor signaling pathway, B cell receptor signaling pathway, T cell receptor signaling pathway, Wnt signaling pathway, chemokine signaling pathway, apoptosis, Jak-STAT signaling pathway, epidermal growth factor receptor (EGFR) signal pathway, mTOR signal pathway, and TGF-beta signaling pathway, which are critical cellular pathways related to virus infection.

**Figure 7:**
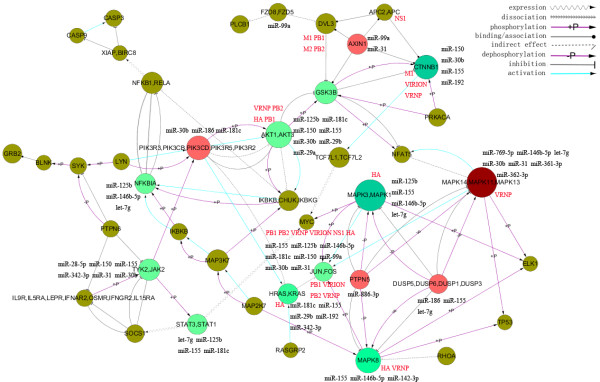
**Influenza virus-related regulatory network.** KEGG pathway analysis was performed on cellular genes identified as important for influenza virus replication. The interactions between nodes in these enriched KEGG pathways were summarized to construct a combined pathway network. The degree level of each gene is represented by the size of the node. The color gradient of the green nodes, from light to dark, shows the value of in-degree of the receivers, from low to high; meanwhile, the color gradient of the red nodes, from light to dark, shows the value of out-degree of the regulators, from low to high. The nodes with degree more the 3 in the combined network were showed in the core sub-network. MiRNAs were located near their targets, and so were the virus proteins which were represented by red fonts.

Among these cellular genes, we summarized the interactions between nodes in these enriched KEGG pathways to construct a combined pathway network. Topological analysis was then performed to determine which nodes can be major regulators and receivers. A major regulator is defined as a node that exerts control over at least five other nodes, whereas a major receiver is influenced by a minimum of five nodes. The nodes with a degree of more than 3 in the combined network were selected to form a subnetwork for further analysis, in which we added the data of miRNAs who have targets validated by previous studies or predicted by a large number of algorithms on the major regulators and receivers. With the additional data of virus-host interactions, we were able to construct Figure 7.

Our data suggest that miRNA dysregulation in the PBMCs of H1N1 critically ill patients can regulate a number of key genes in the major signaling pathways associated with influenza virus infection.

## Discussion

MiRNAs have been reported to participate in regulating cross-talk between the host and the pathogen in viral infections, which have a major function in viral pathogenesis [12-14]. Cellular miRNAs can also be involved in regulating the molecular pathways of innate and adaptive immune responses, and can act as an antiviral defense mechanism or even inhibit virus replication directly [58,59]. Cellular miRNAs can be used by viruses for their own advantage. For example, the hepatitis C virus (HCV) replication is dependent on cellular miR-122 expression. The HCV RNA genome contains two miR-122 binding sites in its 5′UTR, which are required to activate viral genomic RNA replication. Increased miR-122 expression can result in regulating anti-apoptotic genes and enhancing viral replication to promote cell proliferation [60-62].

In our study, we used PBMC cell samples from critically ill patients with H1N1 influenza and identified numerous differentially expressed miRNAs (Figure 1). QRT-PCR assay and ROC curve analyses revealed that miR-31, miR-29a and miR-148a all had significant potential diagnostic value for critically ill patients infected with H1N1 influenza virus, which yielded AUC of 0.9510, 0.8951 and 0.8811, respectively (Figures 3 and 5). Some of these differentially expressed miRNAs via in silico analysis targeted mRNAs of several key genes, including TP53, CASP3, JAK2, IL-10, MX1, TGFBR1, and MAPK14. These changes affect numerous other genes and regulators of metabolism and signaling pathways. These subset gene changes are crucial to H1N1 infection and are responsible for disease progression.

MiR-29a and miR-29b were reported to be downregulated in lung tissues from mice infected with reconstructed 1918 or a nonlethal seasonal influenza virus, Tx/91 [17]. This was consistent with our result. Both miR-29a and miR-29b could repress IFN-gamma production by direct targeting of both T-box transcription factor T-bet and Eomesodermin (Eomes), two transcription factors known to induce IFN-gamma production [63]. Therefore, the downregulated miR-29 may regulate the T helper 1 (Th1) cell differentiation to secrete more IFN-gamma and mediate elimination of intracellular pathogens, but dysregulated T cell responses may also contribute to pathologic inflammation.

E. K. Loveday et al. demonstrated that miR-29a, miR-29c and let-7g were down-regulated in human A549 cells infected with swine-origin influenza pandemic H1N1 [21]. This was consistent with our result. Let-7g could inhibit lectin-like oxidized low-density lipoprotein receptor-1 expression and inhibits apoptosis, by which may suggest increased cell apoptosis [64]. Moreover, let-7g could inhibit the expression of IL-13, a key inducer of airway inflammation secreted by TH2 lymphocytes and other cells [65,66]. Therefore, down-regulation of miR-29a, miR-29c and let-7g may contribute to the uncontrolled inflammation by allowing up-regulation of pro-inflammation genes.

The critically ill patients in this study all had no underlying diseases including type 2 diabetes, immunodeficiency or cardiopulmonary diseases, but they had comorbidities like pneumonia or acute respiratory distress syndrome (ARDS), which may lead to disease progression. We collected samples as soon as patients were admitted to ICU with confirmed influenza A H1N1 infection, when they were very severe and immediately treated with anti-infective therapy and so on. Interestingly, we found all the critically ill patients in our study were overweight (BMI > 25 kg/m^2^). Many reports support the view that obesity is associated with higher risks of ICU admission and death in patients with influenza A (H1N1) infection [67-69]. Other findings suggest that obese patients with severe infection were more likely to develop pneumonitis compared to non-obese patients [70-72]. Infection with influenza virus in diet-induced obese mice was shown to dysregulate immune response, expecially impair the T cell memory response, and lead to increased morbidity and mortality from viral infection [73-75]. A recent study reported that the expression of miR-146b-5p was decreased in monocytes during obesity [76]. MiR-146b-5p acts as an inhibitor of NF-κB-mediated inflammation and is necessary for the anti-inflammatory action of high levels of globular adiponectin. Another group found that let-7g was downregulated in the fetal muscle of diet-induced obese ovine compared to control. The downregulation of let-7g may enhance intramuscular adipogenesis during fetal muscle development in the setting of maternal obesity [77]. Taken together, our findings suggest the downregulation of miR-146b-5p and let-7g were important in further understanding the molecular mechanisms implicated in obese patients susceptive to severe infection of H1N1 influenza virus.

Schmidt et al. [78] found that miR-146b-5p, miR-150, miR-342-3p and let-7g were downregulated in peripheral blood leukocytes during acute lipopolysaccharide (LPS) induced inflammation, which was similar to our result. Several genes encoding proteins involved in NF-κB and MAPK signaling as well as cytokine pathways and other inflammation pathways were predicted targets of these LPS-responsive miRNAs. These miRNAs may play an important role in controlling the level of inflammatory response. A predisposition for pneumococcal infections after H1N1 influenza virus infection has been reported [79]. *Streptococcus pneumonia* co-infection is correlated with the morbidity and the mortality of H1N1 pandemic influenza [80]. Therefore, this result is reasonable because most of our patients had pulmonary infections.

The p38 MAPK are a class of MAPKs.kinases. The p38 MAPK pathway is strongly activated by stress, but also has important functions in the immune response and in regulating cell survival and differentiation, which allows cells to interpret a wide range of external signals and respond appropriately by generating a large number of different biological effects [81]. Studies have shown that influenza virus infection activates MAPK family members in mammals, and the expression of RANTES, IL-8, and tumor necrosis factor-alpha were controlled by p38 activation [82]. P38 MAPK is a determinant of virus infection, which depends on MyD88 expression and Toll-like receptor 4 (TLR4) ligation, and the inhibition of p38 MAPK signaling significantly inhibits virus replication [83]. However, in our study, MAPK14 mRNA expression in critically ill patients had no significant change compared with healthy controls, indicating that the response and the regulation of key gene expression for survival in H1N1 critically ill patients is highly complex. P38 MAPKs (MAPK11, MAPK13, and MAPK14) were found to be regulated by miR-769-5p, miR-146b-5p, let-7g, miR-30b, miR-31, miR-361-3p, and miR-362-3p (Figure 7), which were all down expressed in H1N1 critically ill patients. Thus, increasing the expression of miRNAs targeting p38 MAPKs in H1N1 critically ill patients can help inhibit virus replication. These miRNAs can have an antiviral function during influenza virus infection.

We found that EGFR was regulated by miR-342, miR-155, miR-30b, miR-210, miR-192, let-7g, and miR-146b-5p, which were all down expressed in H1N1 critically ill patients. EGFR can promote the uptake of influenza viruses into host cells by forming a lipid raft-based signaling platform with sialic acids and other receptor tyrosine kinases (RTKs) [84]. These downregulated miRNAs can upregulate EGFR expression, resulting in easier virus replication and propagation at the early stage of infection. This result is additionally supported by that of a recent siRNA screening study, which identified the fibroblast growth factor receptors 1, 2, and 4 as RTKs involved in the early stages of viral infection [6]. The downregulation of this kind of miRNAs helps to regulate the host antiviral response or to benefit the virus by allowing virus replication.

Apoptosis is a hallmark event observed in infection with numerous viral pathogens, including influenza A virus [85]. Sequential activation of caspases can have a central function in the execution phase of cell apoptosis. CASP3 is a major virus-induced apoptosis effector, which can be activated by CASP9 (Figure 7). A previous study showed that the presence of inhibitor that blocks CASP3 or knock-down of CASP3 by siRNAs can significantly impair influenza virus propagation, proving the importance of CASP3 activation for efficient influenza virus replication during the onset of apoptosis [85]. In our study, CASP3 was significantly upregulated by qRT-PCR analysis (Figure 6) and targeted by the downregulated miRNAs: miR-342-3p, miR-29b, miR-29c, miR-29a, let-7g and miR-30b, which can be expected to develop miRNA-based therapeutics for influenza disease.

Transforming growth factor beta (TGF-beta) is a family of proteins secreted by virtually all cells. TGF-beta levels increase during viral infection, and significant TGF-beta levels activated by influenza virus exist to induce cell apoptosis [86]. In our study, TGF-beta receptor 1 (TGFBR1) was found to be downregulated (Figure 6). TP53 is a well-known tumor suppressor that responds to diverse cellular stresses to regulate target genes that induce cell cycle arrest, apoptosis, and senescence. TP53 was also found to be downregulated. A response mechanism of host cell possibly exists to remit apoptosis induced by influenza virus. Moreover, TGFBR1 and TP53 were both predicted to be regulated by high-expressed miR-148a.

We found that miR-148a was significantly upregulated compared with the control samples by qRT-PCR assay, indicating that miR-148a has an important function in influenza virus infection. MiR-148a has been associated with different types of cancer [87,88] and autoimmune diseases, such as multiple sclerosis [23], asthma [89] and systemic lupus erythematosus [90]. A recent study has demonstrated that miR-148a expression is also upregulated in DCs on maturation and activation induced by TLR3, TLR4, and TLR9 agonists, which, in turn, inhibit the upregulation of MHC class II expression, the production of cytokines including IL-12, IL-6, TNF-alpha, and IFN-beta, and antigen presentation of DCs by directly targeting Calcium/calmodulin-dependent protein kinase II [91]. Their result indicates that miR-148a is a negative regulator of the innate response and antigen presenting capacity of DCs. The upregulated miR-148a in PBMCs of H1N1 critically ill patients may contribute to the regulation of innate and adaptive immune responses.

Our miRNA microarray and RT-PCR analysis revealed that miR-31 was significantly down-expressed in PBMCs of H1N1 critically ill patients. MiR-31 can negatively regulate FOXP3 expression by binding directly to its potential target site in the 3′UTR of FOXP3 mRNA [92]. Foxp3+ T regulatory (Treg) cells have an important function in inducing and maintaining immunological tolerance [93]. FoxP3+ Treg cell was significantly increased among H1N1- infected patients compared with normal controls by flow cytometry analysis [79,94]. The inverse correlation between miR-31 expression and Treg cell number in the PBMC of H1N1 critically ill patients can be explained by the negative regulation of FOXP3 expression.

Mx1 protein was proven highly important for long-term protection against influenza virus infection [95-97]. Recently, Cilloniz et al. found that Mx1+/+ mice can generate a protective antiviral response by controlling the expression of key modulator molecules associated with influenza virus lethality [98]. In our study, we found that Mx1 mRNA was significantly upregulated in H1N1 critically ill patients by qRT-PCR assay. No validated miRNA targeting Mx1 has been reported; thus, our miRNA target prediction result indicated that Mx1 can be negatively regulated by miR-342-3p and miR-210, which were both down expressed in H1N1 critically ill patients. Therefore, increasing the Mx1 expression by inhibiting these two miRNAs can enhance protection against influenza virus infection.

Adopting a global perspective is important when investigating infections. A systems biology approach to infectious disease research, which models various interacting component networks, will permit greater understanding of the molecular mechanism and the interplay between the host and pathogen. In our study, with integrated various information (miRNA targets, miRNA-mediated pathways, virus proteins, et al.), we obtained a combined network of core information related to H1N1 infection. A better understanding of the network of genes and cellular pathways regulated by these miRNAs will undoubtedly enable us to characterize the host antiviral mechanism comprehensively and to find new targets for developing antiviral compounds.

Although the results of our study can lead to understanding further the functions of miRNAs in influenza virus infection, additional experiments, such as miRNA target validation, in vivo western blot, and pull-down assays during infection and larger cohort of patients clinical investigation are still needed to validate and to refine our observations.

## Conclusions

We identified the systematic differences in miRNA expression patterns between PBMCs from H1N1 critically ill patients and healthy controls. Using RT-PCR analysis, we verified nine important differentially expressed miRNAs and validated seven core genes. ROC curve analyses revealed that miR-31, miR-29a and miR-148a all had significant potential diagnostic value for critically ill patients infected with H1N1 influenza virus, which yielded AUC of 0.9510, 0.8951 and 0.8811, respectively. In addition, we found that a number of genes and signaling pathways that are important to influenza virus infection are likely to be regulated, at least partly, by miRNAs. Finally, we constructed an influenza virus-related miRNA-mRNA regulatory network, which can lead to a global perspective for investigating influenza virus infection.

Therefore, further understanding the functions of these miRNAs in influenza virus infection will provide new insight into the host-pathogen interactions and pathogenesis.

## Abbreviations

miRNA: MicroRNA; PBMC: Peripheral blood mononuclear cell; WHO: World health organization; RNAi: RNA interference; DC: Dendritic cell; NK: Natural killer; ICU: Intensive care unit; CK: Creatine kinase; CK-MB: Creatine kinase isoenzyme; CDC: Center for prevention and disease control; BMI: Body mass index; TP53: Tumor protein p53; MAPK14: Mitogen-activated protein kinase 14; JAK2: Janus kinase 2; CASP3: Caspase 3 apoptosis-related cysteine peptidase; IL10: Interleukin 10; TGFBR1: Transforming growth factor beta receptor 1; Mx1: Myxovirus resistance 1; EGFR: Epidermal growth factor receptor; ROC: Receiver operating characteristic; AUC: The areas under the ROC curve; BMI: Body mass index

## Competing interests

All authors declare that they have no competing interests.

## Authors’ contributions

Conceived and designed the experiments: HS QW DZ JC. Performed the experiments: HS QW YG. Analyzed the data: HS YG LD. Contributed reagents/materials/analysis tools: SL XG RS BL. Wrote the paper: HS YG QW DZ JC. All authors read and approved the final manuscript.

## Pre-publication history

The pre-publication history for this paper can be accessed here:

http://www.biomedcentral.com/1471-2334/13/257/prepub
